# APLN promotes hepatocellular carcinoma through activating PI3K/Akt pathway and is a druggable target

**DOI:** 10.7150/thno.34713

**Published:** 2019-07-09

**Authors:** Huarong Chen, Chi-Chun Wong, Dabin Liu, Minnie Y.Y. Go, Bin Wu, Sui Peng, Ming Kuang, Nathalie Wong, Jun Yu

**Affiliations:** 1 Institute of Digestive Disease and Department of Medicine and Therapeutics, State Key laboratory of Digestive Disease, Li Ka Shing Institute of Health Sciences, CUHK Shenzhen Research Institute, The Chinese University of Hong Kong, Hong Kong SAR.; 2 Department of Gastroenterology, The Third Affiliated Hospital of Sun Yat-Sen University, Guangzhou, 510630, China; 3 Precision Medicine Institute, First Affiliated Hospital, Sun Yat-sen University, Guangzhou, 510080, China; 4 Department of Liver Surgery, First Affiliated Hospital, Sun Yat-sen University, Guangzhou, 510080, China; 5 Department of Anatomical and Cellular Pathology, The Chinese University of Hong Kong, Shatin, Hong Kong, China

**Keywords:** Hepatocellular carcinoma, APLN, prognosis, PI3K/Akt.

## Abstract

**Background**: The pathogenesis of hepatocellular carcinoma (HCC) is a multistep process contributed by the accumulation of molecular alterations. We identified Apelin (APLN) as an outlier gene up-regulated in hepatocellular carcinoma (HCC) through RNA-Seq and microarray analysis. We aimed to investigate its function, mechanism of action and clinical implication in HCC.

Methods: Gene expression and clinical implication of APLN were assessed in multiple human HCC cohorts. Ectopic expression and silencing of APLN were performed to determine its function. The therapeutic potential of APLN and its downstream pathway was investigated using *in vitro* and *in vivo* models.

**Results**: APLN overexpression was commonly observed in more than 80% of HCCs and independently predicted poorer survival of patients in three independent HCC cohorts. Apelin up-regulation was mediated by active β-catenin, which binds to the APLN promoter to induce transcription. Ectopic APLN expression in HCC cells promoted cell proliferation, accelerated G1/S progression and inhibited apoptosis, whilst APLN knockdown exerted opposite effects *in vitro* and inhibited HCC xenograft growth in mice. Mechanistically, APLN activated phosphatidylinositol 3-kinase/protein kinase B (PI3K/Akt) pathway via APLN receptor, leading to increased expression of phospho-glycogen synthase kinase 3β (p-GSK3β) and cyclin D1. Pharmacological targeting of APLN by ML221 was safe and effective in inhibiting APLN-PI3K/Akt cascade and HCC growth *in vitro* and *in vivo*.

**Conclusions**: Our findings unraveled an oncogenic role of APLN in HCC, and that targeting of APLN might be a promising for HCC treatment. APLN may serve as an independent prognostic factor for HCC patients.

## Introduction

Hepatocellular carcinoma (HCC) is the most common primary liver malignancy, which accounts for 700,000 annual deaths globally in recent years 1. There are numerous known risk factors that contribute to HCC, such as chronic hepatitis B virus (HBV) or hepatitis C virus infections, alcohol abuse, obesity and non-alcoholic fatty liver disease (NAFLD) 2. Although HCC is a clinically and biologically heterogeneous malignancy, most HCCs share common features regarding genetic and epigenetic alterations. Recent studies have highlighted multiple molecular pathways that contribute to the initiation and development of HCC, including receptor tyrosine kinase 3, PI3K/Akt/mTOR 4, Wnt/β-catenin 5, ubiquitin/proteasome degradation 6 and angiogenic pathways 7. Targeting these critical pathways hold promise for improving the long-term outcome of patients. However, the precise molecular events underlying HCC development remain unclear.

Identification of common molecular alterations in HCC might provide a rational strategy for the development of effective molecular targeted therapy in HCC. Using gene expression & transcriptome analysis, we identified Apelin (APLN) as an outlier gene highly expressed in both NAFLD- and HBV-associated HCC compared to their respective adjacent non-tumor tissues. APLN is located on chromosome Xq26.1 and encodes an adipokine prepropeptide that could be cleaved by endopetidases to generate several bioactive carboxy-terminal fragments such as apelin-13, -17, -36 and others 8, 9. Apelin peptides are endogenous ligands for seven-transmembrane G protein-coupled receptor (GPCR) APJ (APLNR) 10. The APLN-APLNR system plays a critical role in normal physiological processes, including angiogenesis 11, energy metabolism 12, gastrointestinal function and fluid homeostasis 13. Moreover, APLN-APLNR signaling is involved in the regulation of multiple human diseases, including heart disease, diabetes, obesity, and cancer 14. In cancer, the APLN-APLNR signaling has been reported to promote maturation of tumor vasculature 15-17. Overexpression of APLN and APLNR has been shown to contribute to arteriogenesis In HCC 18. However, whether APLN has a direct role in the pathogenesis of HCC remains unknown. In this study, we demonstrated that APLN is frequently up-regulated in primary HCC and predicts poor prognosis for HCC patients. APLN plays an oncogenic role in hepatocarcinogenesis by activating the phosphatidylinositol 3-kinase/protein kinase B (PI3K/Akt) pathway. Pharmacological targeting of APLN is effective in suppressing PI3K/Akt pathway and the growth of HCC cells *in vitro* and *in vivo*, indicating that APLN is a promising drug target for HCC treatment.

## Methods

### Human HCC Samples

For discovery cohort (our Hong Kong cohort), 10 pairs of human HBV-related HCC and adjacent normal tissues, and 17 pairs of human NAFLD-related HCC and adjacent non-tumor tissues were collected in Prince of Wales Hospital, the Chinese University of Hong Kong and RNA-Seq was performed in our previous study 19, 20. In addition, 16 and 17 pairs of HCC and adjacent normal tissues for reverse transcription PCR and Western blot were further collected from Prince of Wales Hospital, respectively. Three validation cohorts with large sample size were included in this study. Our Guangzhou HCC cohort consists of surgically excised HCC and adjacent non-tumor tissues from 80 patients (80 HCC and 76 adjacent normal tissues) from the third affiliated hospital of Sun Yat-Sen university, Guangzhou. Specimens were immediately snap frozen in liquid nitrogen and then stored at -80 °C until further processing. Patients were followed up regularly (median time: 23.4 months). Informed consent was obtained for all patients and this study was approved by ethics committee of the Chinese University of Hong Kong and the third affiliated hospital of Sun Yat-Sen University. For the TCGA HCC cohort, APLN mRNA expression (369 HCC and 50 adjacent normal tissues) and patient information (330 with survival data) were acquired from the UCSC Cancer Genomics Browser (https://genome-cancer.ucsc.edu/). For GSE76427 cohort, APLN mRNA expression (115 HCC and 52 adjacent normal tissues) and patient information (115 with survival data) were acquired from GEO (https://www.ncbi.nlm.nih.gov/gds). For GSE9843 cohort, APLN mRNA expression (in 91 HCC) and CTNNB1 mutation status (available in 87 patients) were also acquired from GEO (https://www.ncbi.nlm.nih.gov/gds). The clinicopathological features of patients were shown in **Table S1-4**.

### Cell culture

Hep3B, HepG2, PLC5 and SK-Hep1 were purchased from American Type Culture Collection (ATCC, Manassas, VA). Huh6 and Huh7 were purchased from Japanese Collection of Research Bioresources Cell Bank (JCRB, Japan). Normal human liver cell lines (MIHA and LO2) were kindly provided by Dr Alfred Sze-Lok Cheng from the Chinese University of Hong Kong 21, 22. These cells were cultured in DMEM (Invitrogen, Carlsbad, CA). NAFLD-HCC cell lines (HKCI-2 and HKCI-10) were kindly provided by Dr Natalie Wong from the Chinese University of Hong Kong 19, 20, 23 and cultured in RPMI1640 medium (Invitrogen). All medium were supplemented with 10% Fetal Bovine Serum (Thermo Scientific, Waltham, MA) and cells were maintained at a 37 °C in a humidified incubator with 5% CO_2_.

### Real-time quantitative PCR

Total RNA was extracted from cells and tissues using Trizol reagent (Life Tech, Carlsbad, CA). cDNA was synthesized from total RNA using High-Capacity RNA-to-cDNA Kit (Life Tech). Quantitative RT-PCR was performed using a LightCycler 480 real-time PCR system (Roche Applied Sciences, Indianapolis, IN) using Light-Cycler 480 SYBR Green I Master Mix (Roche) following the manufacturer's instructions. The primers used were listed in **Table S5**. Gene expression was normalized to β-actin and calculated using 2-ΔΔCt Method.

### Construction and Transfection of Expression Vectors

The pcDNA3.1 expression vector containing full-length open reading frames of human APLN (NM_017413.4) was constructed. The sequence of the construct was confirmed. Cells were transfected with pcDNA3.1-APLN or empty vector using lipofectamine 2000 (Life Tech). Stably transfected cells were established by selection with neomycin (G418) (Life Tech). Transient transfection of constitutively active β-catenin (S33Y) or empty vector was performed as described previously 24.

### Small Interfering RNA-Mediated Gene Silencing

The sequences of CTNNB1-siRNAs (Genepharma) are as follows:

CTNNB1-siRNA: GCUCAUCAUACUGGCUAGUTT (sense: 5'-3') and ACUAGCCAGUAUGAUGAGCTT (anti-sense: 5'-3'). A non-targeting RNA sequence served as a negative control. APLNR-specific siRNAs (sc-44732) was purchased from Santa Cruz. Cells were transfected using Lipofectamine 2000 (Invitrogen).

### Lentivirus Packaging and Transduction

Two shRNAs targeting APLN (shAPLN-1: 5'- TCTCCCATAAGGGACCCAT-3' and shAPLN-2: 5'- GGGCCTGACATGGCTATAT-3') and a non-targeting RNA sequence serving as a negative control were cloned into the pGPU6 vector (Genepharma, Shanghai, China). Virus packaging was performed in HEK293T cells.

### Cell Viability and Colony Formation Assay

For cell viability assays, cells (1x10^3^ per well) were seeded in a 96-well plate and MTT assay (5 mg/ml; Promega, Madison, WI) was performed according to the manufacturer's protocol. For colony formation assay, cells (500 per well) were seeded in 12-well plate. After 2 weeks, colonies were fixed and stained with crystal violet. PI3K inhibitors BYL-719 and GDC-0941 were from ApexBio (Shanghai, China). Specific APLN receptor inhibitor ML221 was purchased from R&D Systems (Minneapolis, MN) 25. These drugs were added at indicated concentrations.

### Cell Cycle Analysis

Cells were serum starved overnight and stimulated with complete medium for 12 to 24 h. Cells were fixed in 70% ethanol, stained with propidium iodide (BD Biosciences, San Jose, CA) and analyzed by flow cytometry. The cell cycle was analyzed by FlowJo software using Dean-Jett-Fox model.

### Apoptosis Assay

Carbobenzoxy-Leu-Leu-leucinal (MG132) (Cell Signaling Technology) and Staurosporine (STS) (Sigma-Aldrich, St. Louis, MO) was used to induce apoptosis. Apoptosis was determined using the Annexin-phycoerythrin/7-aminoactinomycin D staining kit (BD Biosciences).

### Chromatin Immunoprecipitation Assay

According to the Magna ChIP™ protocol (Merck Millipore, Billerica, Mass), Huh7 cells were fixed with 1% formaldehyde at room temperature for 10 min. Glycine was added to cells to quench unreacted formaldehyde for 5 min. Cell pellet in SDS Lysis Buffer containing 1X Protease Inhibitor Cocktail were sonicated at 4 °C to yield fragments from 100 to 500 bps. Immunoprecipitation was performed using anti-β-Catenin (D10A8) antibody (Cell Signaling Technology) and ChIP-grade Protein A/G Magnetic Beads (Merck Millipore). Protein-DNA crosslinks were reversed at 65 °C and DNA was purified for qPCR. The primers used were listed in **Table S5**.

### Western Blot Analysis

Western blot was performed as previously described 26. Briefly, the total protein was loaded to a SDS-PAGE gel (6% stacking gel and 15% separating gel). After gel electrophoresis, the proteins were transferred to a nitrocellulose membrane at 70V for 2 h and then blocked with 5% bovine serum albumin (BSA) for 30 min at room temperature. Next the blocked membrane was incubated with the primary antibody overnight at 4 °C. Primary antibodies used were APLN (sc-33804), APLNR (sc-33823) and β-actin (sc-47778) (Santa Cruz Biotechnology, Santa Cruz, CA); Phospho-PI3 Kinase p85 (Tyr458) (4228), Akt (9272), phospho-Akt (Ser473) (9271), GSK3β (9315), phospho-GSK3β (Ser9) (9336), Cleaved Caspase-3 (9661), Cleaved PARP (5625) and Cyclin D1 (2922) (Cell Signaling Technology, Danvers, MA). Protein quantification was performed by Image J.

### Immunohistochemistry (IHC)

A total of 13 formalin-fixed, paraffin-embedded HCC tissues from surgery were collected in Department of Gastroenterology, the first affiliated hospital of Sun Yat-Sen University, Guangzhou. IHC was performed as previously described 26, 27. Briefly, 4-mm thick sections were cut from paraffin-embedded tissue blocks. Anti-human APLN antibody (sc-33804, 1:50 dilution) was used. The staining score of APLN was determined according to the percentages of positive cells and staining intensity. The results were scored independently by two pathologists. This study was approved by ethics committee of the first affiliated hospital of Sun Yat-Sen University.

### Subcutaneous Xenograft Mouse Models

For subcutaneous xenografts, HCC cells transduced with lentiviral carrying shCTL and shAPLN-1 (or control and APLN-overexpressing HepG2 stable cell lines) were injected subcutaneously into the left and right dorsal flanks of 4-weeks-old male Balb/c nude mice (2×10^6^ cells in 0.1 mL phosphate-buffered saline (PBS)/mice), respectively. Tumor size was measured every 2 days using a digital caliper. Tumor volume (mm^3^) was calculated as follows: volume = (shortest diameter)^ 2^ × (longest diameter) × 0.5. At the end point, tumors were harvested and weighted.

To investigate the effect of ML221 on HCC growth in vivo, PLC5 (3×10^6^ cells) or HepG2 (2×10^6^ cells) cells were injected subcutaneously into dorsal flanks of 4-weeks-old male Balb/c nude mice. ML221 was dissolved in sterile dimethyl sulfoxide (DMSO), diluted to a concentration of 1 mg/mL in normal saline solution, and administered intraperitoneally to the mice (10 mg/kg body weight) every other day. Control mice were injected with vehicle. All animal studies were performed in accordance with the guidelines approved by the Animal Experimentation Ethics Committee of CUHK.

### Luciferase Reporter Assay

Nine cancer pathway-related reporters including BMP, MAPK (SRE), nuclear factor-кB (NF-кB), p38, p53, PI3K/Akt (FHRE), STAT5 (LHRE), STAT3 (APRE) and TGF-β were included in this study 27. Briefly, cells were seeded into a 24-well plate and co-transfected with luciferase reporter and Renilla (internal control) reporter. Two days after transfection, cells were harvested, and the Firefly and Renilla luminescence were measured by the dual-luciferase reporter assay system (Promega). Reporter activity was determined as the ratio of Firefly to Renilla luciferase activity.

### Statistical Analysis

All results were expressed as mean ± SD unless otherwise indicated. To compare the difference between two groups, Mann-Whitney U-test, Wilcoxon matched-pairs test and Student's t-test were performed. The difference between growth rates was determined by ANOVA with repeated-measures analysis of variances. The Pearson chi-square test or Fisher's exact test was used for analysis of the associations between patient clinicopathological characteristics and APLN expression. Kaplan-Meier analysis and log-rank test were performed to evaluate the association between APLN expression and patient survival. Cutoff value of APLN was analyzed by survival significance analysis using the tool Cutoff Finder (http://molpath.charite.de/cutoff/) 28. COX proportion hazard regression model was performed to assess the prognostic value of APLN expression. All statistical tests were performed using Graphpad Prism 5.0 (GraphPad Software Inc., San Diego, CA) or SPSS, version 20.0 (SPSS Inc, Chicago, IL), and a two-tailed P-value of 0.05 was considered statistically significant.

## Results

### APLN is frequently over-expressed in HCC

**Figure 1:**
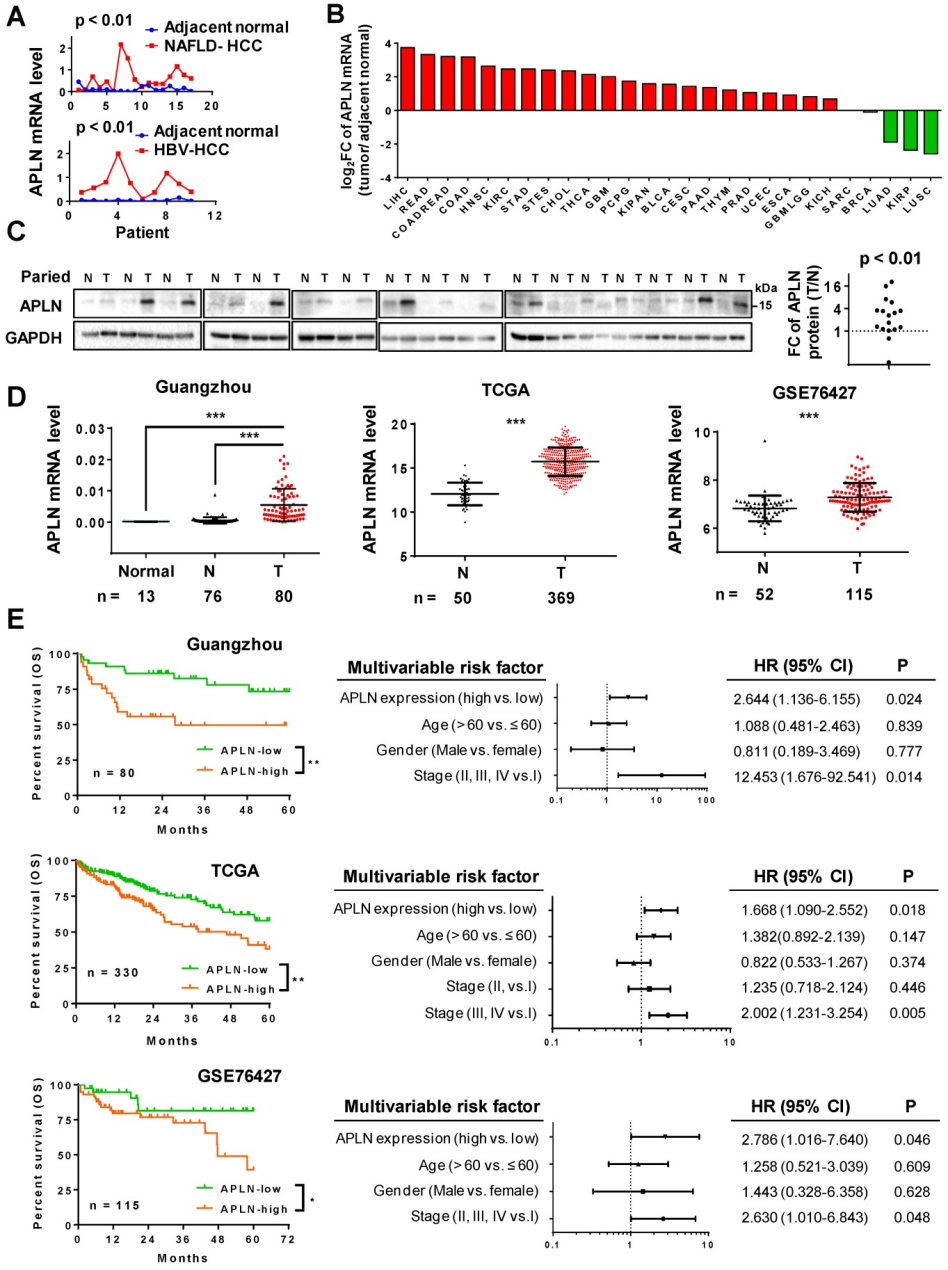
** APLN is overexpressed in HCC tissues and is a prognostic biomarker for HCC patients. (A)** mRNA expression of APLN in 17 and 10 paired HCC and adjacent non-tumor tissues from NAFLD-HCC (RNA-seq) and HBV-HCC patients (gene expression array) from Hong Kong cohort. Wilcoxon matched-pairs test was performed.** (B)** Fold change (FC) of APLN mRNA expression (tumor/ adjacent normal) across multiple cancer types from TCGA. Data was acquired from firebrowse (http://firebrowse.org). Full name of cancer-type abbreviations and their sample sizes are provided in Table S1. **(C)** Protein expression of APLN in 17 pairs of HCC (T) and adjacent normal tissue (N). Quantification was performed by ImageJ. Wilcoxon matched-pairs test was performed. **(D)** mRNA expression of APLN in normal liver tissues (Normal), adjacent normal tissues (N) and HCC (T) from three independent HCC cohorts. Mann-Whitney U-test was performed. **(E)** Kaplan-Meier survival analysis (left panel) indicated that HCC patients with high expression of APLN had worse overall survival in three independent HCC cohorts. By multivariate Cox regression analysis (right panel), high APLN expression was an independent prognostic factor in all three cohorts. (* *P* < 0.05, ** *P* < 0.01, *** *P* < 0.001).

We analyzed the gene expression profiles of HBV-associated HCC (gene expression array) and NAFLD-associated HCC (RNA-seq) (**Figure 1A**). APLN was over-expressed in 9/10 (90.0%) HBV-associated HCC and 14/17 (82.3%) NAFLD-associated HCC. Consistent with our data, TCGA pan-cancer analysis revealed that APLN mRNA is most profoundly up-regulated in HCC among all cancer types (**Figure 1B and Table S1**). Reverse transcription polymerase chain reaction (RT-PCR) (n = 16) and Western blot (n = 17) analysis of paired HCC and adjacent normal tissues verified that APLN was highly up-regulated in tumors at both mRNA and protein levels, respectively (**Figure 1C and S1A**). By immunohistochemical staining of human HCC specimens, APLN was located in the cytoplasm of tumor tissues with higher expression as compared to adjacent normal tissues (*P* < 0.01, **Figure S1B**). Three independent HCC cohorts were further included in this study. In our Guangzhou cohort, APLN mRNA was not detected in the normal liver by quantitative PCR (qPCR). In contrast, APLN mRNA was highly induced in HCC tumors (n = 80) compared to adjacent normal tissues (n = 76) (*P* < 0.001, **Figure 1D**). Consistent results were observed in two other independent HCC cohorts (TCGA and GSE76427) (*P* < 0.001, **Figure 1D**).

### High APLN expression predicts worse survival in HCC patients

The correlation between APLN expression and clinicopathological features of HCC were assessed in three HCC cohorts. APLN mRNA expression was not correlated with clinical parameters including age, gender and TNM staging by ANOVA analysis (**Table S2-4**).

Kaplan-Meier curves revealed that high APLN mRNA expression was significantly associated with worse overall survival in HCC patients from our Guangzhou cohort (n = 80, *P* < 0.01), TCGA cohort (n = 330, *P* < 0.01) and GSE76427 cohort (n = 115, *P* < 0.05) (**Figure 1E**). By multivariate Cox regression analysis, high APLN expression was found to be an independent prognostic factor that predicts shorter overall survival in all three cohorts (*P* < 0.05; **Figure 1E**). Hence, APLN is an adverse prognostic factor in HCC patients.

### APLN expression is induced by Wnt/β-catenin signaling

**Figure 2:**
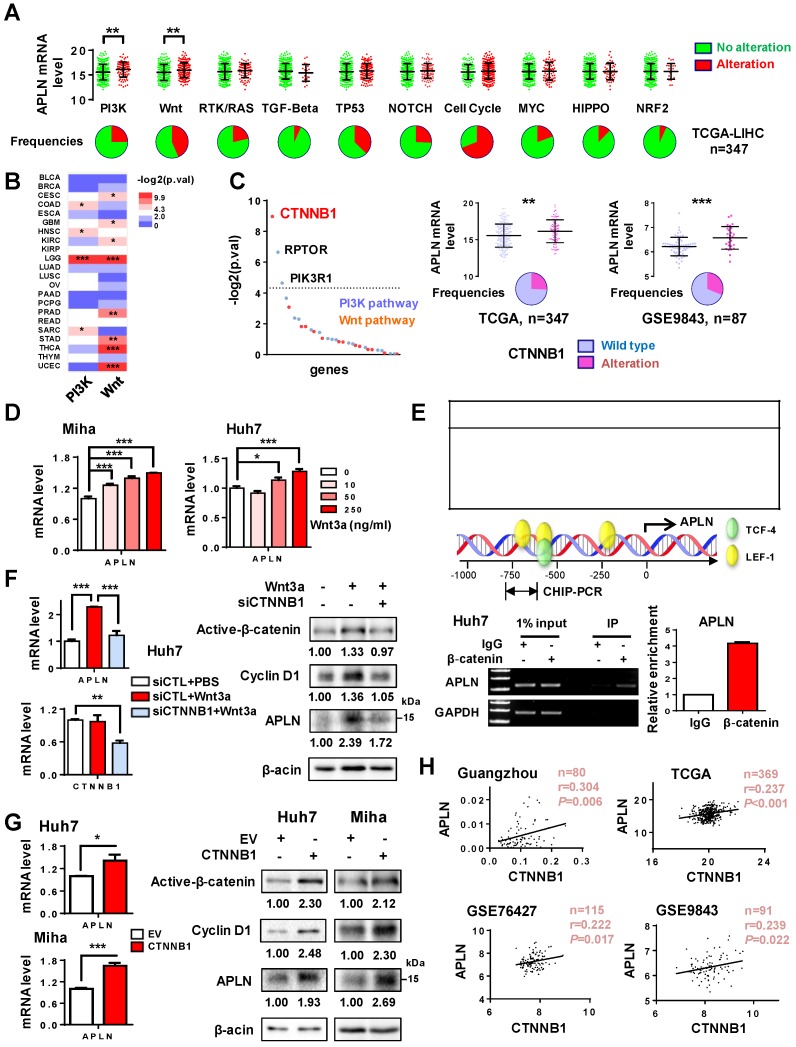
** APLN is a downstream target of WNT/β-catenin signaling. (A)** Alteration frequencies of ten canonical pathways (cell cycle, Hippo, Myc, Notch, Nrf2, PI3K/Akt, RTK-RAS, TGF-β signaling, p53 and Wnt/β-catenin) in TCGA-LIHC cohort were displayed in pie charts. Student's t-test was performed. (**B**) Comparison of APLN expression between tumors with alteration in PI3K/Akt (or Wnt/β-catenin) pathway and those without. Full name of cancer-type abbreviations were provided in Table S1. The p values were obtained by Student's t-test and shown as heatmap. (**C**) In TCGA-LIHC cohort, correlations of APLN expression with individual gene alteration from PI3K/Akt and Wnt/β-catenin pathway were assessed and showed in left panel. Right panel demonstrated that HCC tissues carrying mutant β-catenin exhibited higher APLN expression as compared to those with wild type in both TCGA and GSE9843 cohorts. Student's t-test was performed. (**D**) Treatment of Miha and Huh7 cells with recombinant human Wnt3a protein for 24h promoted the transcription of APLN in a dose-dependent manner. (**E**) Top panel showed in silico promoter prediction, which identified binding sites for TCF-4/LEF-1 in promoter region (-1kb to +100bp) of APLN. Middle panel indicated predicted binding sites for TCF-4/LEF-1 (PROMO), and the promoter regions analyzed by ChIP-PCR. Bottom panel revealed the interaction of β-catenin with APLN promoter by chromatin-immunoprecipitation PCR and qPCR analysis. (**F**) Wnt3a-induced APLN expression was blocked by β-catenin knockdown both at mRNA (left panel) and protein (right panel) level in Huh7 cell. (**G**) Transient transfection of constitutively active β-catenin (S33Y) promoted mRNA (left panel) and protein (right panel) expression of APLN both in Huh7 and Miha cells. (**H**) CTNNB1mRNA expression is positively correlated with APLN mRNA expression in Guangzhou, TCGA, GSE76427, and GSE9843 cohorts. Error bars in D-G represent means ± sd from three independent experiments, (* *P* < 0.05, ** *P* < 0.01, *** *P* < 0.001).

Numerous mechanisms contribute to gene up-regulation in cancer, including DNA amplification, genetic mutation, gene rearrangement or gene activation 29. APLN was only infrequently mutated in HCC patients (1/373) in TCGA cohort. In addition, no significant correlation was found between its DNA copy number and mRNA expression (**Figure S2A**). We thus hypothesized that up-regulation of APLN in HCC may be attributed to activation of its transcription factor(s). We first analyzed the correlation of APLN expression with deregulation of ten canonical cancer pathways including cell cycle, Hippo, Myc, Notch, Nrf2, PI3K/Akt, RTK-RAS, TGFβ, p53, and Wnt/β-catenin in TCGA HCC cohort. Alterations of these cancer pathways were defined by the genetic alterations (activating or inactivating events) of their candidate pathway members 30. If one or more genes in this pathway inside the tumor contained a recurrent or known driver alteration, the tumor was considered as altered in a particular pathway. We found that APLN mRNA expression was significantly increased among HCC tumors with alterations in PI3K/Akt or Wnt/β-catenin pathways (**Figure 2A**), an observation that was validated in other tumor types (**Figure 2B**). Among the genes from these two pathways, β-catenin (CTNNB1) was the top outlier associated with the induction of APLN in HCC (**Figure 2C**). In both TCGA and GSE9843 cohorts, HCC harboring activating β-catenin mutations exhibited higher APLN expression compared to those with wildtype β-catenin (**Figure 2C**). Similar trends were observed for the target genes of Wnt/β-catenin (AXIN2, BMP4, CCND1, LEF1 and MYC) (**Figure S2B**). To evaluate whether APLN is a downstream target of Wnt/β-catenin signaling, Miha and Huh7 cells were treated with recombinant human Wnt-3a protein, a Wnt agonist that activates canonical Wnt signaling. As shown in **Figure 2D**, Wnt-3a significantly promoted the transcription of APLN in a dose-dependent manner. Abnormal activation of Wnt/β-catenin signaling leads to the accumulation of β-catenin in the nucleus, where it binds to T-cell factor/lymphoid enhancer factor (TCF/LEF) transcription factors and activates transcription of downstream target genes. We next performed *in silico* promoter prediction using three independent databases, which unanimously identified TCF/LEF transcription factor binding sites in the promoter region of APLN (-1kb to +100bp) (**Figure 2E**). Using chromatin-immunoprecipitation PCR assay, we confirmed the direct binding of β-catenin to the APLN promoter (**Figure 2E**). Furthermore, silencing of β-catenin in Huh7 cells abolished the induction of APLN by Wnt-3a at both mRNA and protein levels (**Figure 2F**). On the contrary, ectopic expression of activated β-catenin significantly enhanced the mRNA and protein expression of APLN in both Huh7 and Miha cells (**Figure 2G**). Cyclin D1, a target gene of Wnt/β-catenin, was included as positive control (**Figure 2F-G**). Our results imply that β-catenin binds to the promoter region of APLN and regulates its expression. In addition, significant positive correlation between mRNA expression of APLN and CTNNB1 was observed in four different HCC cohorts (**Figure 2H**). Together, these data indicate that APLN is a target of aberrant Wnt/β-catenin signaling in HCC.

### APLN promotes cell proliferation, accelerates cell cycle and inhibits apoptosis

**Figure 3:**
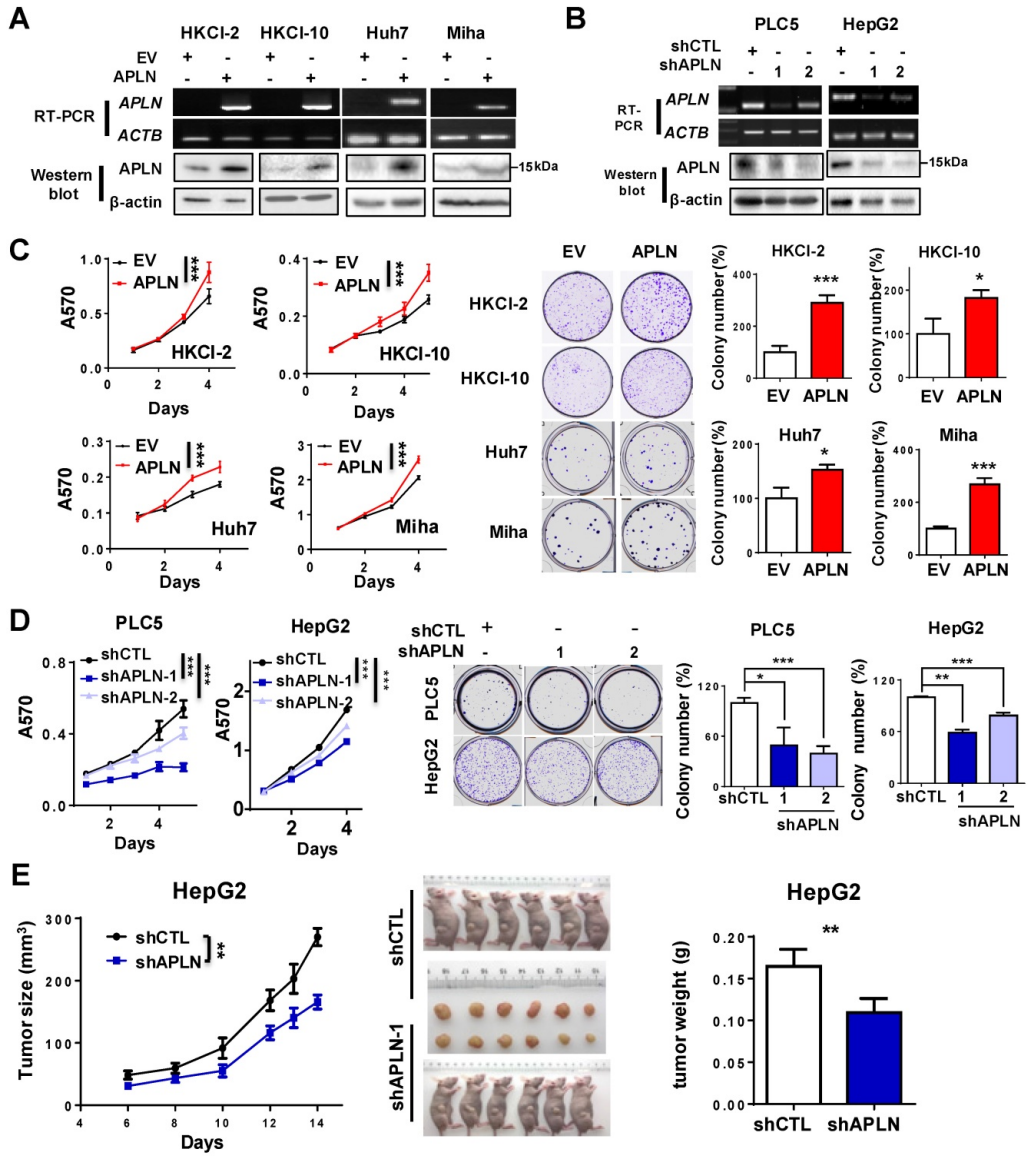
** APLN promotes cells proliferation. (A)** HKCI-2, HKCI-10, Huh7 and Miha were stably transfected with pcDNA3.1 expression vector carrying APLN cDNA or empty vector (EV). Ectopic expression of APLN was confirmed by RT-PCR (Top panel) and western blot analysis (Bottom panel). **(B)** PLC5 and HepG2 were transduced with lentiviral carrying APLN-specific shRNAs (shAPLN) or negative control shRNA (shCTL). Knockdown of APLN was confirmed by RT-PCR (Top panel) and western blot analysis (Bottom panel).** (C)** Ectopic expression of APLN significantly promoted cell viability by MTT assay and increased colony numbers compared to controls.** (D)** Knockdown of APLN significantly reduced cell viability by MTT assay and decreased colony numbers in PLC5 and HepG2 compared to controls. **(E)** Knockdown of APLN suppressed the growth of HepG2 xenografts in nude mice (N = 6) compared to controls. Representative images of HepG2 xenografts and tumor weight were shown. Error bars in C and D represent means ± sd from three independent experiments, (* *P* < 0.05, ** *P* < 0.01, *** *P* < 0.001).

APLN protein expression was assessed in multiple liver cancer cell lines and one immortalized liver epithelial cell line (MIHA) and four normal human liver tissues by western blot (**Figure S3A**). APLN protein was readily expressed in all liver cancer cell lines, whilst no or very low expression was observed in normal liver tissues or MIHA cells (**Figure S3A**). To determine the functional role of APLN, we overexpressed APLN in HKCI-2, HKCI-10, Huh7 and MIHA cells with low APLN expression, while silenced APLN with two independent APLN-specific shRNAs in PLC5 and HepG2 cells that exhibit high APLN expression. Ectopic expression or silencing of APLN was confirmed by RT-PCR and western blot analysis (**Figure 3A-B**). Overexpression of APLN significantly increased cell viability and clonogenicity in HKCI-2, HKCI-10, Huh7 and MIHA cells (**Figure 3C**). Conversely, silencing of APLN reduced cell proliferation and colony formation in PLC5 and HepG2 cells (**Figure 3D**).

To further investigate the pro-tumorigenic effect of APLN, HepG2-shCTL and HepG2-shAPLN-1 cells were subcutaneously injected into the left and right dorsal flanks of nude mice, respectively.

As shown in **Figure 3E**, knockdown of APLN significantly suppressed tumor growth in nude mice (*P* < 0.05). The mean tumor weight of HepG2-shAPLN-1 group was decreased compared to HepG2-shCTL group (**Figure 3E**,* P* < 0.05). Consistent result was obtained in the PLC5 xenograft model (**Figure S3B**). On the contrary, overexpression of APLN promoted the growth of HepG2 xenografts in nude mice (**Figure S3C**).

**Figure 4:**
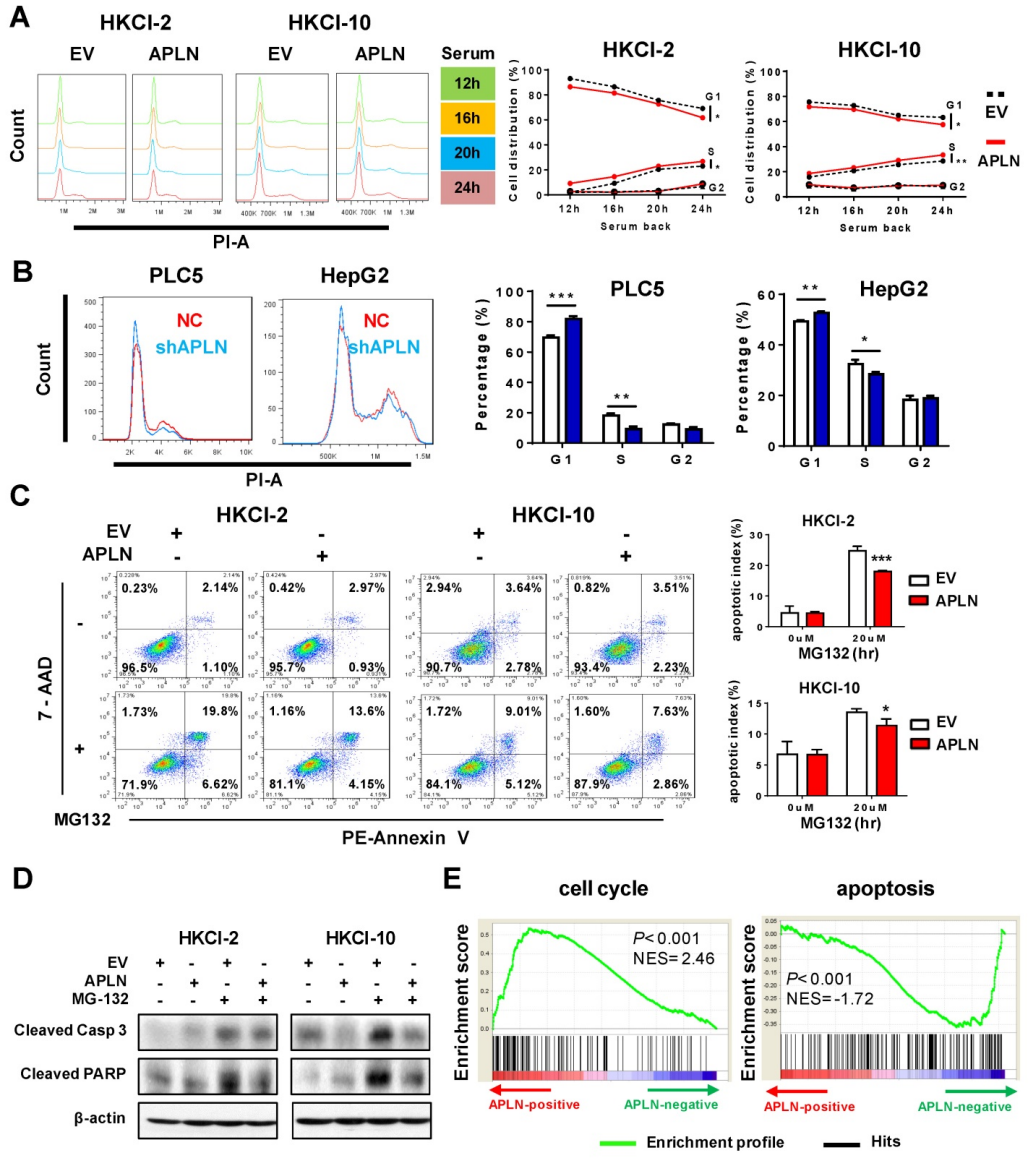
** APLN accelerates cell cycle progression and inhibits apoptosis. (A)** Up-regulation of APLN accelerated G1 to S phase cell cycle transition. The difference of serum-induced cell cycle progression between control cells and cells overexpressing was determined by ANOVA with repeated-measures analysis of variances. **(B)** APLN knockdown induced G1-phase cell cycle arrest in PLC5 and HepG2 cells. Student's t-test was performed. **(C)** HKCI-2 and HKCI-10 cells were treated with 20uM MG132 for 24h. Apoptosis was analyzed by flow cytometry. **(D)** After MG132 treatment, the expression of cleaved caspase-3 and PARP was determined by western blot. **(E)** Gene set enrichment analysis for correlation of APLN with all other genes from HCC samples of TCGA-LIHC cohort. NES, normalized enrichment score. Error bars in B and C represent means ± sd from three independent experiments, (* *P* < 0.05, ** *P* < 0.01, *** *P* < 0.001).

We next examined effect of APLN on cell cycle and apoptosis. Cell cycle analysis showed that overexpression of APLN in HKCI-2, HKCI-10 and Huh7 cells accelerated cell cycle progression from G1 to S phase compared to controls (**Figure 4A and S4A**). Conversely, knockdown of APLN induced G1-phase cell cycle arrest in PLC5 and HepG2 cells (**Figure 4B**). Furthermore, HKCI-2 and HKCI-10 cells overexpressing APLN were more resistant to MG132-induced apoptosis as compared to controls (**Figure 4C**), concomitant with the decreased activation of apoptosis markers cleaved caspase-3 and PARP (**Figure 4D**). Consistent data were obtained in cells treated with another apoptosis inducer, STS (**Figure S4B**). Conversely, knockdown of APLN induced apoptosis in PLC5 and HepG2 cells (**Figure S4C**). Gene set enrichment analysis (GSEA) of RNA-seq data of 371 HCC samples from TCGA cohort indicated a positive correlation between APLN expression and cell cycle, but a negative correlation between APLN expression and apoptosis pathways (**Figure 4E**). Thus, APLN exerted its oncogenic function by promoting cell cycle progression and rendering cells resistant to apoptosis induction.

### APLN activates PI3K/Akt signaling

**Figure 5:**
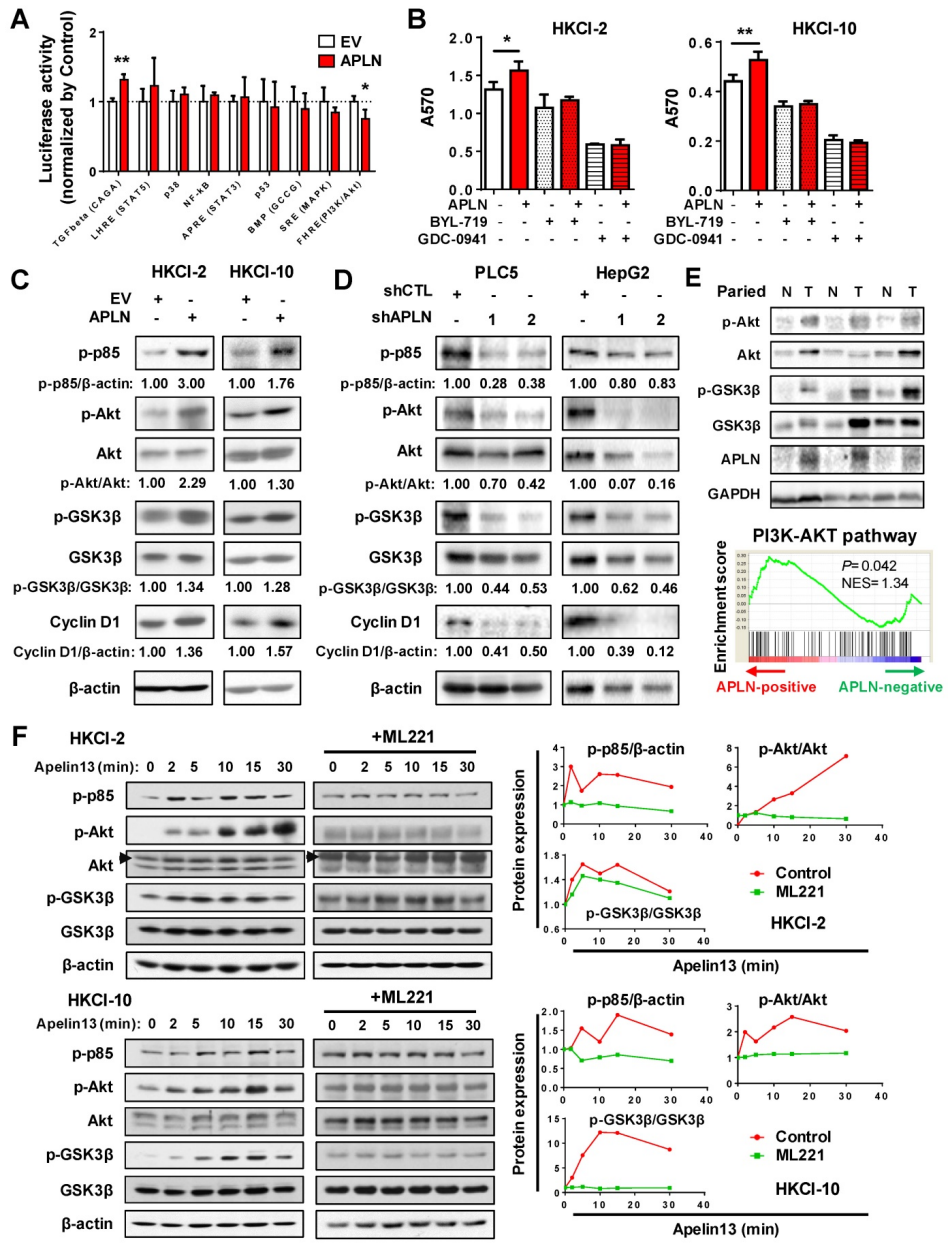
** APLN activates PI3K/Akt signaling. (A)** Nine cancer pathway- related firefly luciferase reporter constructs were co-transfected with renilla luciferase reporter (internal control) in HKCI-10 cells. **(B)** BYL-719 (4uM) and GDC-0941 (1uM), two specific PI3K/Akt Inhibitors, were added to HKCI-2 or HKCI-10 cells. Control cells were treated with an equivalent dilution of DMSO. MTT assays were performed. **(C)** Upregulation of APLN increased phospho-PI3K p85, phospho-Akt, phospho-GSK3β and Cyclin D1 expression by western blot analysis.** (D)** APLN knockdown decreased the expression of phospho-PI3K p85, phospho-Akt, phospho-GSK3β and Cyclin D1 by western blot analysis.** (E)** Top panel showed APLN overexpression was associated with the increased expression of phospho-Akt and phospho-GSK3β in human HCC specimens. Bottom panel demonstrated positive correlation of APLN with PI3K-AKT pathway in TCGA-LIHC cohort by gene set enrichment analysis. **(F)** HKCI-2 and HKCI-10 cells were serum starved overnight and stimulated with 1uM Apelin-13 for indicated time. For ML221 treatment, HKCI-2 and HKCI-10 cells were pretreated with 50µM ML221 for 30min before Apelin-13 stimulation. Cell lysates were blotted with indicated antibodies. The images are representative of two independent experiments. Error bars in A and B represent means ± sd from three independent experiments, (* *P* < 0.05, ** *P* < 0.01, *** *P* < 0.001).

To gain insights into the molecular mechanisms underlying the pro-tumorigenic action of APLN, we screened nine important cancer pathways (BMP, MAPK (SRE), nuclear factor-кB (NF-кB), p38, p53, PI3K/Akt (FHRE), STAT3 (APRE), STAT5 (LHRE) and TGF-β by luciferase reporter assays. Ectopic expression of APLN in HKCI-10 cells specifically induced PI3K/Akt signaling, as evidenced by reduced FOXO reporter activity (*P* < 0.05; **Figure 5A**), an effect validated in MIHA cells (**Figure S5A**). Conversely, APLN knockdown suppressed PI3K/Akt pathway (**Figure S5B**). To test whether the oncogenic function of APLN depends on PI3K/Akt activation, HCC cells with or without ectopic expression of APLN were treated with PI3K/Akt inhibitors BYL-719 and GDC-0941. Both BYL-719 and GDC-0941 treatment effectively inhibited Akt phosphorylation (**Figure S5C**), and markedly abolished growth promoting effect of APLN (**Figure 5B**), indicating that APLN promotes HCC by activating PI3K/Akt pathway. APLN increased phospho-PI3K p85, phospho-Akt, phospho-glycogen synthase kinase-3beta (GSK3β) and Cyclin D1 expression (**Figure 5C**). Conversely, depletion of APLN had opposite effects on PLC5 and HepG2 cells (**Figure 5D**). Correspondingly, induction of APLN expression was correlated with up-regulation of phospho-Akt and phospho-GSK3β in human HCC tissues (**Figure 5E**). Moreover, GSEA of RNA-seq data from TCGA HCC cohort revealed a positive correlation between APLN expression and PI3K/Akt pathway (**Figure 5E**). To investigate the direct effect of APLN on PI3K/Akt signaling, HKCI-2 and HKCI-10 cells were treated with an APLN peptide, Apelin-13. Our results demonstrated that Apelin-13 induced phosphorylation of PI3K p85, Akt and GSK-3β in a time-dependent manner (**Figure 5F**). Apelin is endogenous ligand for APLNR. We next checked the expression of APLNR in paired HCC tissues from patients, normal liver cell lines and HCC cell lines by western blot. The results showed that APLNR was expressed in all tissues and cell lines investigated (**Figure S5D-E**). It was worth noting that manipulating APLN expression affected neither mRNA nor protein expression of APLNR (**Figure S5F-G**). Moreover, the positive effect of apelin-13 on PI3K/Akt signaling was abolished by ML221, a specific APLN receptor antagonist (**Figure 5F**). Taken together, the oncogenic effect of APLN in hepatocarcinogenesis is mediated, at least in part, by the activation of PI3K/Akt signaling.

### APLN-APLNR is a potential therapeutic target of HCC

**Figure 6:**
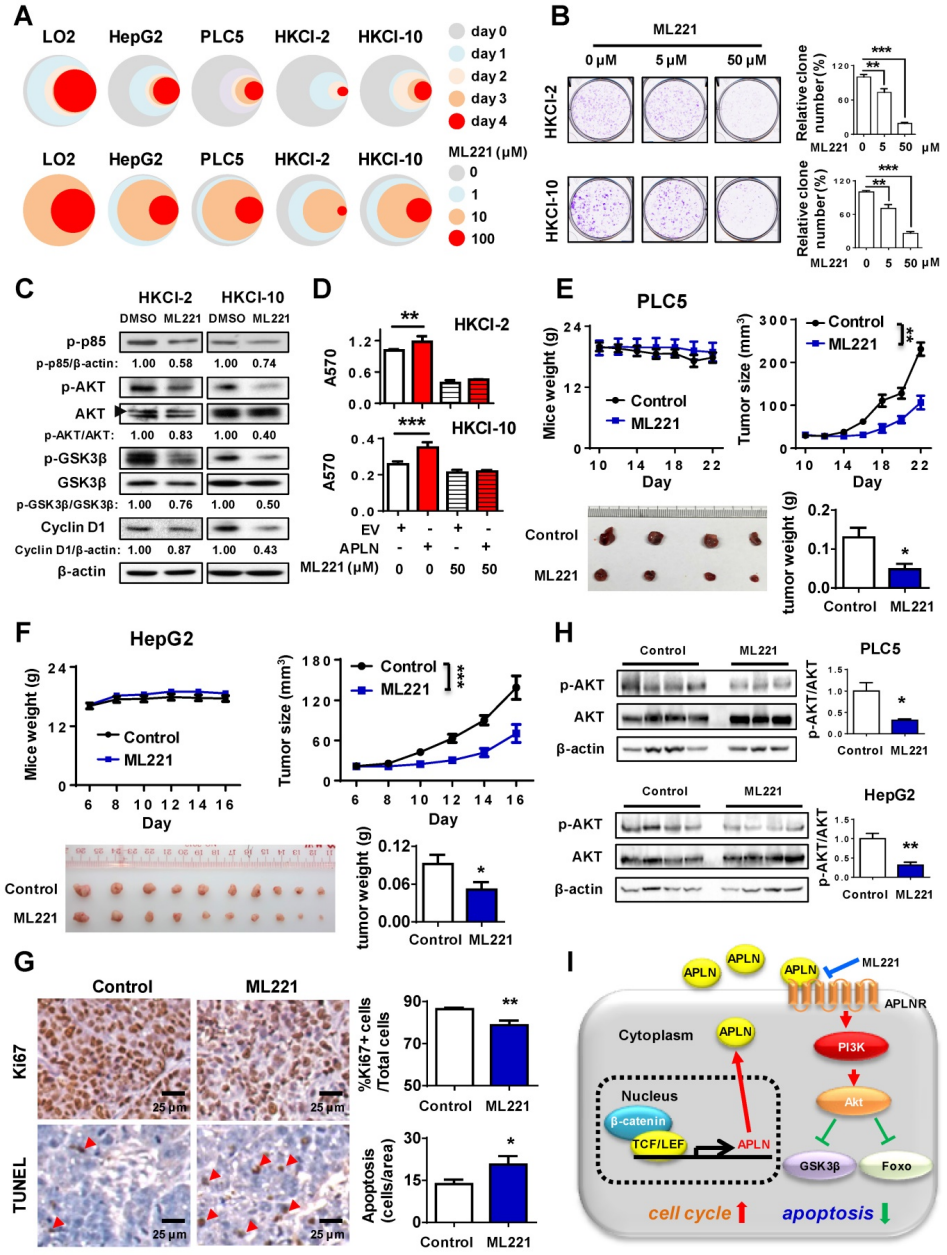
** APLN-APLNR is a potential therapeutic target of HCC.** (**A**) One immortalized hepatocyte cell lines (LO2) and four HCC cell lines (HepG2 PLC5, HKCI-2 and HKCI-10) were treated with ML221 and cell viability was assessed by MTT assay. Top panel: Cells were treated with 100 µM ML221. The colors represent different time points and the diameter indicates relative cell viability. Bottom panel: Cells were treated with different concentrations of ML221 for three days. The colors represent different ML221 concentration and the diameter indicates relative cell viability. (**B**) ML221 treatment significantly decreased colony numbers in HKCI-2 and HKCI-10 cells in a dose-dependent manner. (**C**) HKCI-2 and HKCI-10 cells were treated with 50 µM ML221 for 24h. Cell lysates were then blotted with indicated antibody. (**D**) ML221 (50 µM) was added to HKCI-2 or HKCI-10 cells. Cell viability was assessed by MTT assay. (**E and F**) Intraperitoneal injection of ML221 (10 mg/kg, every other day) significantly inhibited the growth of subcutaneous PLC5 xenografts (N = 4) (**E**) and HepG2 xenografts (N = 10) (**F**), as evidenced by reductions in tumor volume and weight. ML221 treatment had no effect on body weight. (**G**) Representative images of Ki67-positive (Top panel) and TUNEL-positive cells (Bottom panel). At least 4 fields per slide and 2 slides per tumor were counted at 200 × magnification. (**H**) Western blot analysis of PLC5 xenografts (Top panel) and HepG2 xenografts (Bottom panel) confirmed that ML221 treatment inhibited PI3K/Akt signaling, as evidenced by the decreased expression of phospho-Akt. Error bars in B and D represent means ± sd from three independent experiments, (* *P* < 0.05, ** *P* < 0.01, *** *P* < 0.001).

To assess the therapeutic implication of APLN, we treated LO2, HepG2, PLC5, HKCI-2 and HKCI-10 cells with ML221, a competitive antagonist of APLN receptor. ML221 significantly inhibited the proliferation of multiple HCC cell lines (HepG2, PLC5, HKCI-2 and HKCI-10) in a time- and dose- dependent manner (**Figure 6A**). As compared to normal liver cell LO2, HCC cells are more sensitive to ML221 treatment (**Figure 6A**). To assess the specific and cytotoxic effects of ML221 on HCC cells, we further treated Miha, HepG2 and PLC5 with 50 μM ML221 for three different time intervals (day 0, 2 and 3). Consistently, ML221 at 50 μM was non-toxic against normal liver cell line Miha, but significantly suppressed the growth of HCC cell lines HepG2 and PLC5 (**Figure S6A**). ML221 also effectively inhibited colony formation of HKCI-2 and HKCI-10 cells in a dose-response manner, without affecting APLN expression (**Figure 6B and S6B**). Cell cycle analysis demonstrated that ML221 treatment induced cell cycle arrest at G1 phase (**Figure S6C**). In addition, ML221 treatment significantly inhibited PI3K/Akt pathway, as evidenced by the decreased expression of phospho-PI3K p85, phospho-Akt, phospho-GSK3β and Cyclin D1 (**Figure 6C**). Importantly, ML221 treatment abolished the growth advantage rendered by APLN overexpression (**Figure 6D**), implying involvement of APLN-APLNR interaction in the oncogenic effect of APLN. To verify that ML221 targets APLNR on tumor cells, we silenced APLNR in HepG2 and PLC5 cells using siRNAs. The results demonstrated that silencing of APLNR abolished the suppressive function of ML221 on HCC cells (**Figure S6D**). To evaluate the therapeutic effect of ML221 on HCC in vivo, we administered ML221 intraperitoneally to nude mice harboring subcutaneous HCC xenografts. Since HKCI-2 and HKCI-10 cells failed to grow tumors *in vivo*, PLC5 and HepG2 cells were investigated. ML221 was effective in suppressing the growth of HCC xenografts, as evidenced by significant reductions in tumor volume and tumor weight (**Figure 6E-F**). ML221 treatment was well-tolerated as there was no significant difference in the body weight between control and treatment groups (**Figure 6E-F**). ML221 suppressed cell proliferation but induced apoptosis in HepG2 xenografts, as indicated by Ki-67 and TUNEL assays, respectively (**Figure 6G**). Moreover, ML221 treatment significantly inactivated Akt signaling in both PLC5 and HepG2 xenografts (**Figure 6H**). Collectively, our data indicate that APLN is a potential druggable target for HCC.

## Discussion

In this study, gene expression & transcriptome analysis led to the discovery of APLN, an oncogenic factor highly expressed in HCC. APLN is a transcriptional target of Wnt/β-catenin pathway and mediates its oncogenic effect by activating PI3K/Akt signaling, thereby promoting hepatocarcinogenesis. Pharmacological targeting of APLN robustly suppressed proliferation of HCC cells as well as the growth of tumor xenografts, implying that APLN might be a potential drug target for HCC.

APLN gene encodes a 77-amino-acid prepropeptide originally identified from the bovine stomach 9. APLN is synthesized as a preproapelin that undergoes proteolytic maturation to generate bioactive carboxy-terminal fragments such as apelin-13, -17 and -36 31, which bind to and activate the APLNR, a formerly orphan G-protein coupled receptor 10. Among them, apelin-13 is the most widely studied one that exhibits the highest activity at the receptors 32. APLN has been reported to be involved in the regulation of cancer 14. Up-regulation of APLN has been shown in brain 33, colon 34, gastroesophageal 35 and lung cancers 16. In this study, we demonstrated that APLN is consistently overexpressed in HCC from multiple HCC patient cohorts. TCGA pan-cancer analysis further revealed that up-regulation of APLN is the most profound in HCC among other common cancers, indicating its importance in this specific cancer type. We next investigated the clinical implication of APLN and reported here for the first time that APLN overexpression is an independent predictor for poor survival of HCC patients. APLN is a reliable and consistent prognostic marker that may be useful for stratification of HCC patients according to their outcomes.

APLN-APLNR signaling has been implicated in cancer. However, how activation of APLN-APLNR regulates tumorigenesis and tumor progression remains unclear. Sorli et al. showed that APLN up-regulation promotes angiogenesis and tumor growth in mouse cancer models 17. APLN is likely to activate tumor neoangiogenesis through paracrine-mediated action 17. In addition, Kidoya et al. reported that APLN-APLNR system stimulates the morphological and functional maturation of blood vessels in tumors 36. In HCC, APLN-APLNR signaling was found to regulate arteriogenesis by promoting arterial smooth muscle cell proliferation 18. Thus tumor-associated neoangiogenesis mediated by APLN-APLNR signaling contributes to cancer development. However, in our study, APLN and APLNR were both expressed in HCC tissues as well as HCC cell lines. In addition, both mRNA and protein expressions of APLN were significantly higher in HCC as compared to adjacent normal tissues. We presumed that APLN up-regulation could exert a direct oncogenic role in HCC. Our result demonstrated for the first time that APLN directly promotes HCC via binding to APLNR, inducing cell cycle progression and suppressing apoptosis. Consistent with our findings, Picault et al. reported an autocrine loop of APLN-APLNR signaling participating in the growth of colon adenocarcinomas 34. Ectopic expression of APLN induces cyclin D1, which accelerates cell cycle transition from G1 to S phase. APLN also inhibits expression of active caspase-3 and PARP, thereby suppressing induction of apoptosis. Conversely, either APLN knockdown or inhibiting APLN-APLNR interaction exerts opposite effects i*n vitro* and significantly reduced growth of HCC xenografts *in vivo*. Our work highlights an important role of APLN-APLNR axis in promoting HCC and that the targeting of APLN-APLNR interaction is a promising therapeutic strategy for HCC treatment.

Pathway screening identifies PI3K/Akt as the major downstream signaling mechanism underlying the oncogenic effect of APLN in HCC. Either the ectopic expression of APLN or exogenous addition of apelin-13 activates PI3K/Akt pathway and induces phosphorylation of PI3K p85, Akt and GSK-3β. These effects are abolished by the APLNR antagonist ML221. Akt is a central hub in PI3K/Akt signaling pathway that coordinates cell response to extrinsic stimuli and its aberrant activation promotes cell proliferation and survival 37. Activated Akt prevents cyclin D1 from degradation mediated by GSK3β 38, 39 and also blocks apoptosis signaling 40, 41, all of which are consistent with the phenotypic effects of APLN. Blockade of PI3K/Akt signaling abrogates the growth advantage rendered by APLN, corroborating involvement of PI3K/Akt in the tumorigenic function of APLN. APLN-mediated PI3K/Akt signaling has important implications for HCC.

Our work also unveiled for the first time that APLN is a target of WNT/β-catenin pathway in HCC. HCC is characterized by the aberrant up-regulation of WNT/β-catenin pathway, driven principally by activating mutations in β-catenin (20-40%) 42-44. Active β-catenin translocates to nucleus where it binds to LEF/TCF to initiate transcription 45. By analyzing the correlation between APLN expression and alteration of molecular events in TCGA HCC cohort, β-catenin is identified as a top candidate associated with APLN expression. We verified that β-catenin binds to APLN promoter and regulates APLN expression. In agreement with our data, β-catenin/PPARγ has been reported to regulate APLN expression at the level of transcription in pulmonary arterial endothelial cells 46. Consistent with these findings, HCC tumors carrying mutant β-catenin have higher APLN expression, and APLN expression is positively correlated with CTNNB1 expression in three independent HCC cohorts, suggesting that WNT/β-catenin pathway activation mediates APLN overexpression in HCC. Collectively, we have identified a novel WNT/β-catenin-APLN/APLNR-PI3K/Akt axis that operates to promote hepato-carcinogenesis. Targeting of APLN-APLNR by ML221 inhibit PI3K/Akt pathway, which in turn suppresses the growth of HCC.

In summary, our study unravels an important role of APLN in promoting HCC tumorigenicity via accelerating cell cycle progression and inhibiting apoptosis, by a mechanism dependent on the activation of PI3K/Akt signaling. APLN is commonly up-regulated in HCC and is an independent poor prognostic factor for HCC patients. Targeting of APLN-APLNR axis holds promise in treatment of HCC.

## Supplementary Material

Supplementary figures and tables.

## Abbreviations

FC: fold change
GPCR: G protein-coupled receptor
GSEA: gene set enrichment analysis
GSK3β: glycogen synthase kinase 3β
HBV: hepatitis B virus
HCC: hepatocellular carcinoma
NAFLD: non-alcoholic fatty liver disease
PI3K/Akt: phosphatidylinositol 3-kinase/protein kinase B
RT-PCR: reverse transcription polymerase chain reaction
qPCR: quantitative PCR
TCF/LEF: T-cell factor/lymphoid enhancer factor
