# Supplementary material for: APLN promotes hepatocellular carcinoma through activating PI3K/Akt pathway and is a druggable target
Source: Theranostics. 2019 Jul 9;9(18):5246–60. doi: 10.7150/thno.34713 (PMC6691573; doi:10.7150/thno.34713)
Supplement: Supplementary file 1 — Supplementary figures and tables. [file thnov09p5246s1.pdf]

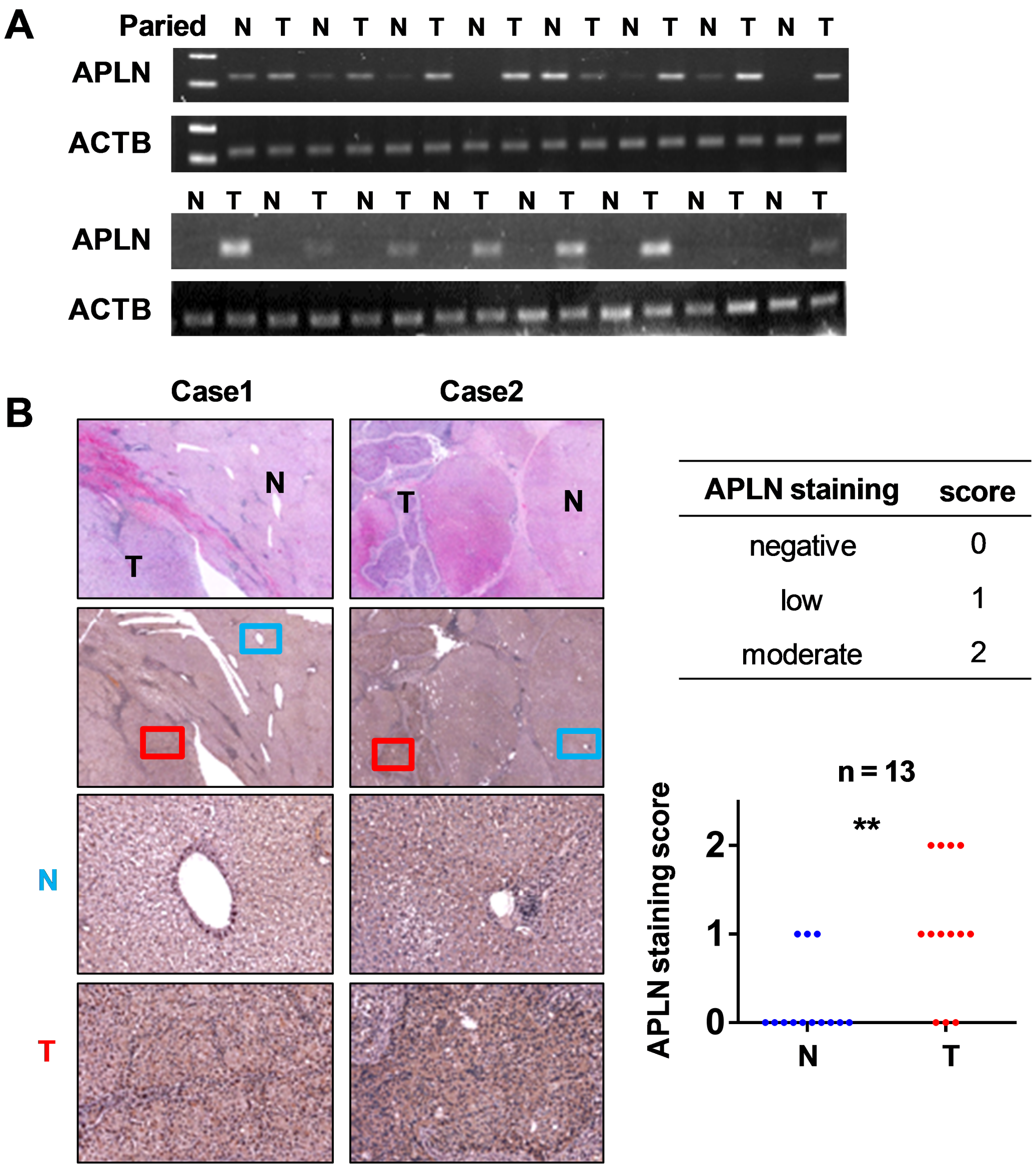

Figure S1. APLN expression is upregulated in HCC. (A) APLN mRNA expression in 16 pairs of HCC (T) and adjacent normal tissues (N) was determined by RT-PCR. **(B)** Left panel showed representative images of APLN protein expression in HCC tissues (T) and adjacent normal tissue (N) of two patients by immunohistochemistry. Right panel showed staining score of APLN in 13 pairs of HCC tissues (T) and adjacent normal tissue (N).

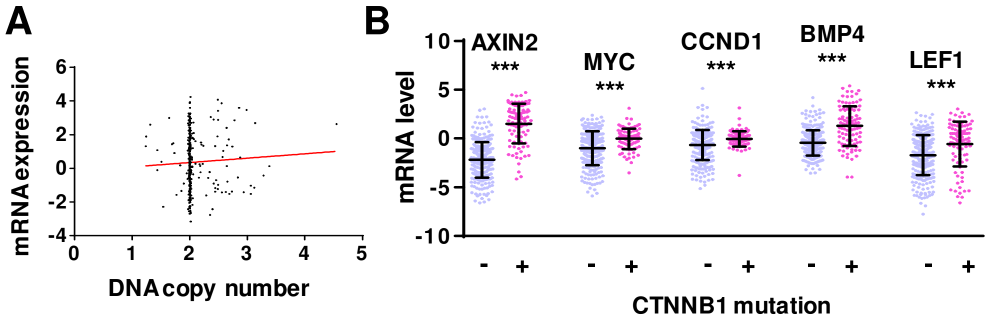

Figure S2. Correlation between mRNA expression of Wnt/β-catenin target genes and β-catenin mutations in HCC. (A) The correlation between DNA copy number and mRNA expression of APLN in 364 HCC from TCGA Cohort. **(B)** The mRNA expression of downstream targets of WNT/β-catenin (AXIN2, MYC, CCND1, BMP4 and LEF1) in HCC with wild type β-catenin (-) and HCC with mutant β-catenin (+) from TCGA. (*** $P < 0.001$).

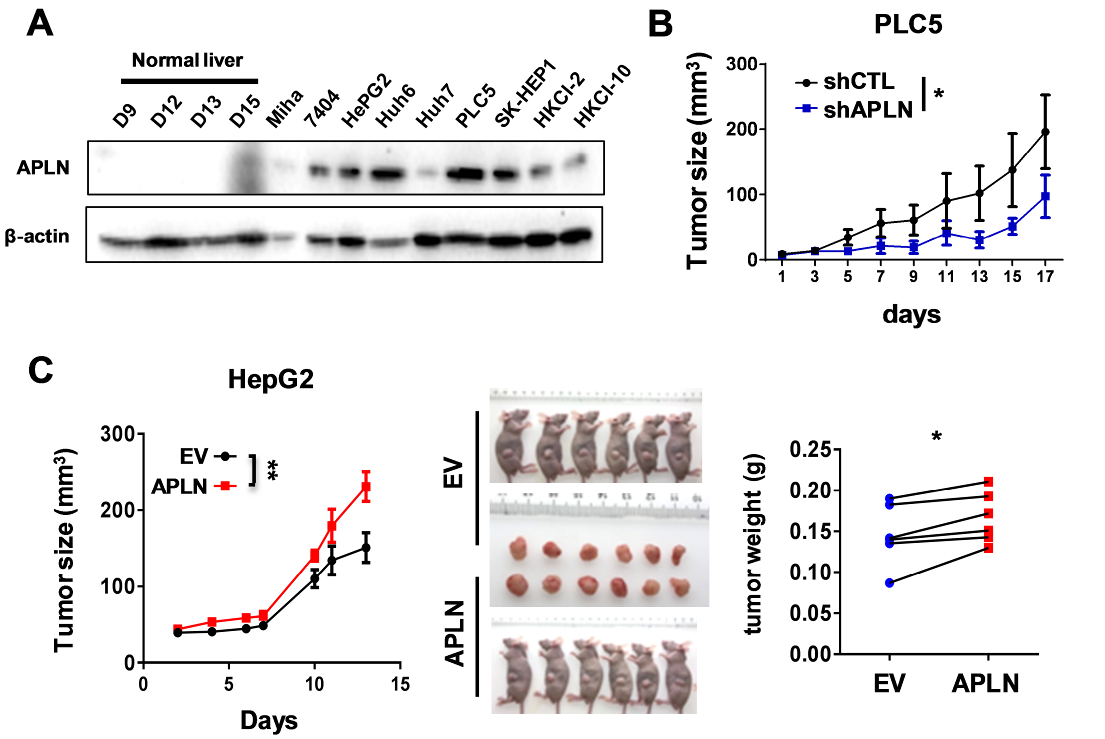

Figure S3. Silencing of APLN reduced HCC tumor growth in vivo. (A) APLN protein expression in liver cancer cell lines (7404, HepG2, Huh6, Huh7, PLC5, HKCI-2 and HKCI-10), one immortalized liver epithelial cell line (MIHA) and four normal human liver tissues. **(B)** Knockdown of APLN suppressed the growth of PLC5 xenografts in nude mice (n = 3) compared to controls. **(C)** Overexpression of APLN promoted the growth of HepG2 xenografts in nude mice (n = 6). Representative images of HepG2 xenografts and tumor weight were shown.

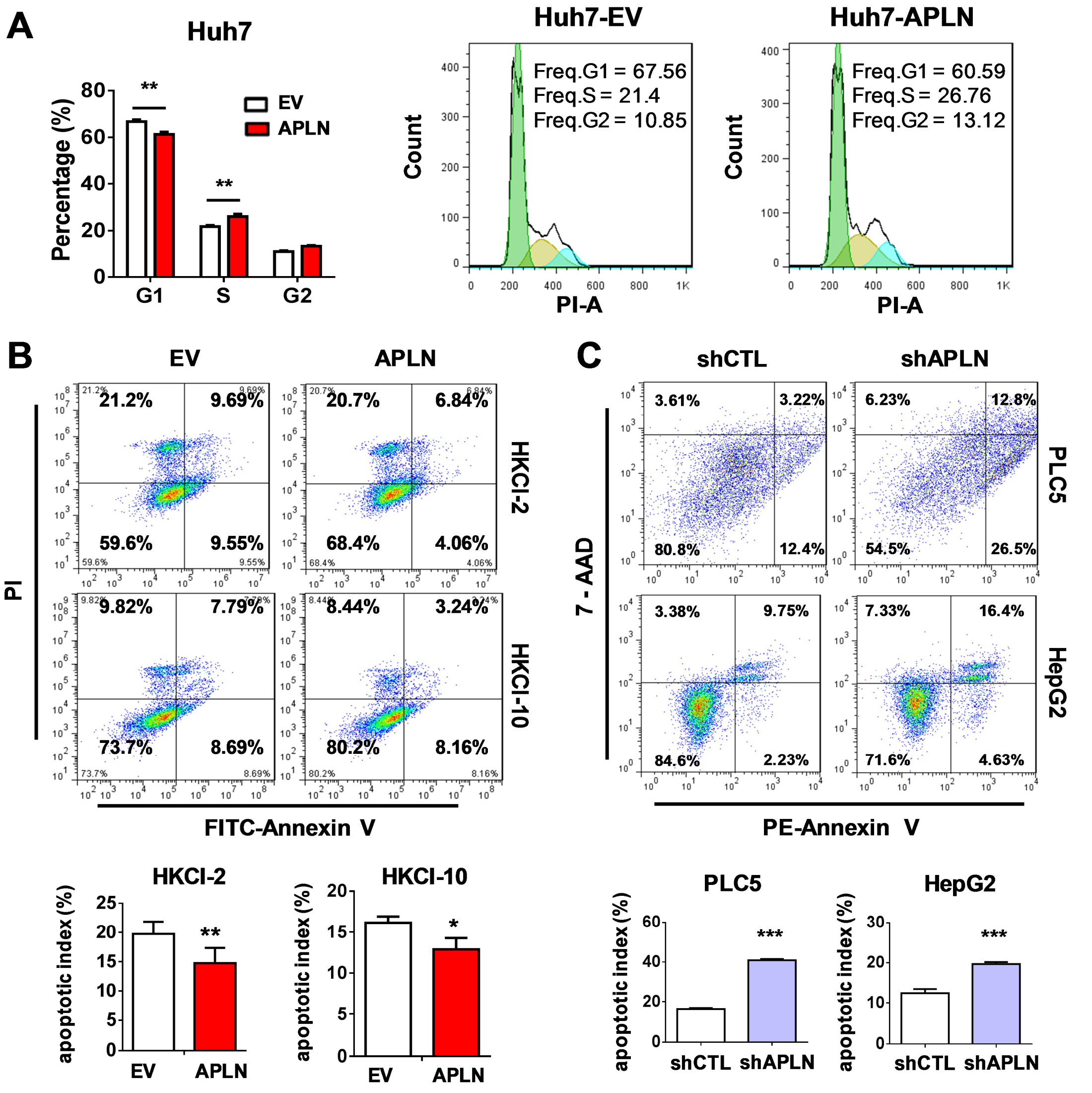

Figure S4. APLN regulates cell cycle and apoptosis. (A) APLN overexpression promoted cell cycle transition in Huh7 cell. **(B and C)** HKCI-2, HKCI-10, PLC5 and HepG2 cells were treated with 1 µM STS for 3h. Apoptosis was analyzed by flow cytometry. The apoptotic index was defined as the percentage of apoptotic cells. (* P < 0.05, ** P < 0.01, *** P < 0.001).

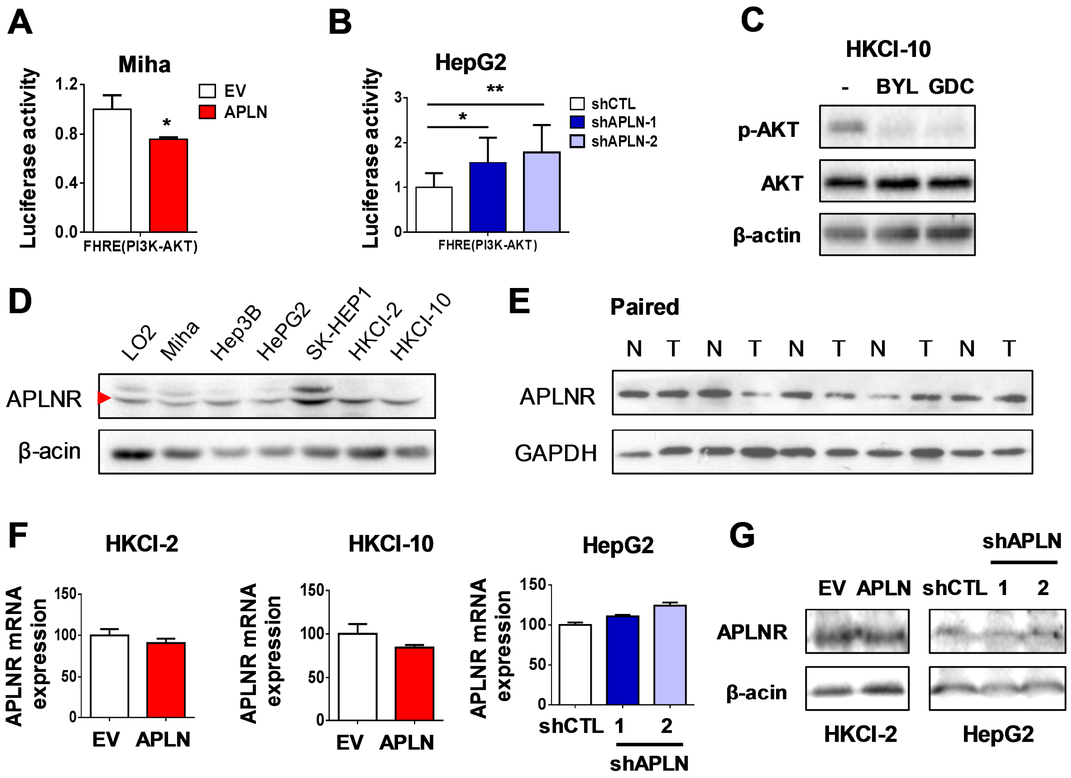

Figure S5. Inhibition of APLN inactivated PI3K/Akt cascade and HCC growth. (A) Ectopic expression of APLN in Miha cells promoted PI3K/Akt signaling as evidenced by reduced FOXO luciferase reporter activity. **(B)** Silencing of APLN in HepG2 cells inhibited PI3K/Akt pathway. **(C)** HKCI-10 cells were treated with 4 µM BYL-719 (BYL) and 1 µM GDC-0941 (GDC) for 1h. The cell lysates were blotted with indicated antibody. **(D)** APLN protein expression in fiver liver cancer cell lines and two immortalized liver epithelial cell line (MIHA and LO2). **(E)** APLNR protein expression in 5 pairs of HCC (T) and adjacent normal tissues (N) was determined by western blot. **(F and G)** Enhancing or reducing APLN expression did not affect either APLNR mRNA (F) or protein expession (G). (* $P < 0.05$, ** $P < 0.01$, *** $P < 0.001$).

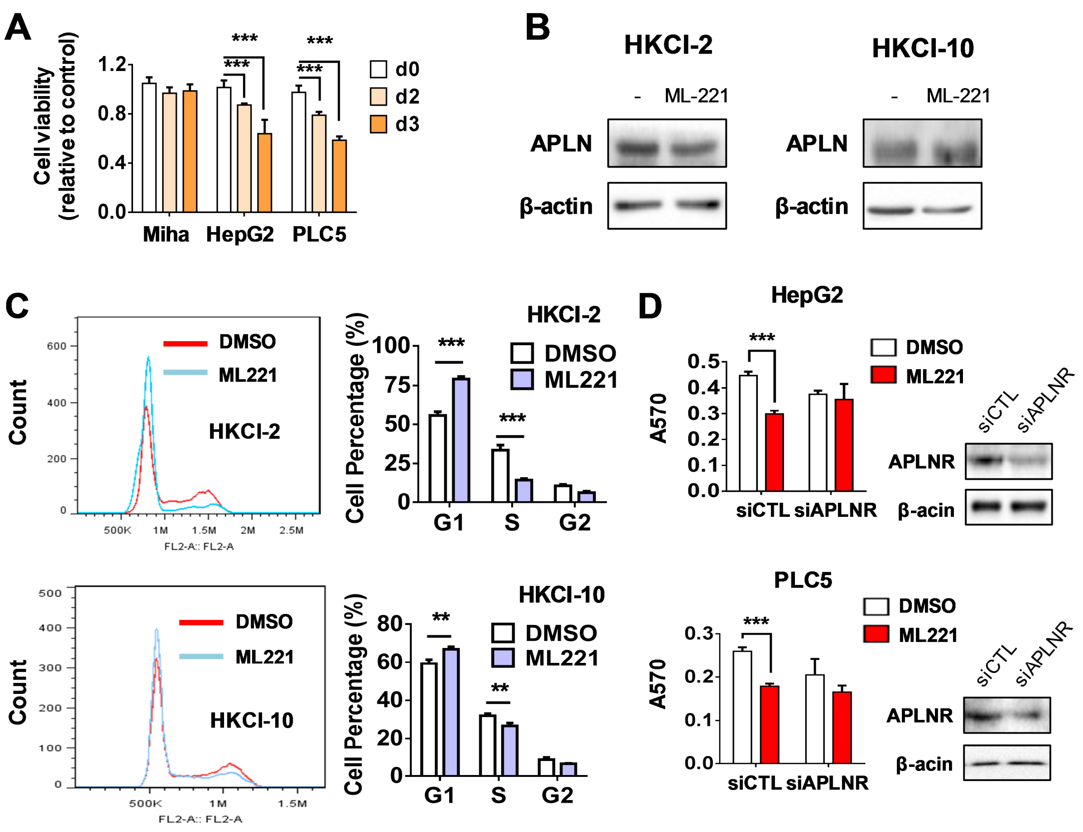

Figure S6. Inhibition of APLN suppressed HCC growth. (A) Miha, HepG2 and PLC5 cells were treated with 50 μM ML-221 for different time intervals (day 0, 2 and 3). Cell viability was assessed by MTT assay. **(B)** HKCI-2 and HKCI-10 cells were treated with 50 μM ML-221 for 24 h. The cell lysates were blotted with indicated antibody. **(C)** HKCI-2 and HKCI-10 cells were serum starved overnight and then stimulated with complete medium containing 50 μM ML221 (DMSO for control group) for 24 h. **(D)** HepG2 and PLC5 cells were transfected with APLNR-specific siRNAs or control-siRNA and then treated with 50 μM ML-221 (DMSO for control group) for 2 days. Cell viability was assessed by MTT assay. (* $P < 0.05$, ** $P < 0.01$, *** $P < 0.001$).

Table S1: Differential expression analysis of APLN across various cancer types in TCGA datasets

Cancer type	Fold change (Tumor vs. Normal)	Sample size (T and N)	Cancer type	Fold change (Tumor vs. Normal)	Sample size (T and N)
**LIHC**	**13.4**	**T:373; N:50**	CESC	2.7	T:306; N:3
READ	9.99	T:167; N:10	PAAD	2.58	T:179; N:4
COADREAD	9.29	T:626; N:51	THYM	2.33	T:120; N:2
COAD	9.02	T:459; N:41	PRAD	2.09	T:498; N:52
HNSC	6.24	T:522; N:44	UCEC	2.05	T:546; N:35
KIRC	5.54	T:534; N:72	ESCA	1.89	T:185; N:11
STAD	5.52	T:415; N:35	GBMLGG	1.77	T:696; N:5
STES	5.28	T:600; N:46	KICH	1.6	T:66; N:25
CHOL	5.12	T:36; N:9	SARC	1.01	T:263; N:2
THCA	4.43	T:509; N:59	BRCA	0.933	T:1100; N:112
GBM	4.04	T:166; N:5	LUAD	0.271	T:517; N:59
PCPG	3.36	T:184; N:3	KIRP	0.194	T:291; N:32
KIPAN	3.01	T:891; N:129	LUSC	0.167	T:501; N:51
BLCA	2.94	T:408; N:19			

BLCA: Bladder urothelial carcinoma; **BRCA**: Breast invasive carcinoma; **CESC**: Cervical and endocervical cancers; **CHOL**:Cholangiocarcinoma; **COAD**:Colon adenocarcinoma; **COADREAD**: Colorectal adenocarcinoma; **ESCA**: Esophageal carcinoma; **GBM**: Glioblastoma multiforme; **GBMLGG**: Glioma; **HNSC**: Head and Neck squamous cell carcinoma; **KICH**: Kidney Chromophobe; **KIPAN**: Pan-kidney cohort (KICH+KIRC+KIRP); **KIRC**: Kidney renal clear cell carcinoma; **KIRP**: Kidney renal papillary cell carcinoma; **LGG**, lower grade glioma; **LIHC**: Liver hepatocellular carcinoma; **LUAD**: Lung adenocarcinoma; **LUSC**: Lung squamous cell carcinoma; **PAAD**: Pancreatic adenocarcinoma; **PCPG**: Pheochromocytoma and Paraganglioma; **PRAD**: Prostate adenocarcinoma; **READ**: Rectum adenocarcinoma; **SARC**: Sarcoma; **SKCM**: Skin Cutaneous Melanoma; **STAD**: Stomach adenocarcinoma; **STES**: Stomach and Esophageal carcinoma; **THCA**: Thyroid carcinoma; **THYM**: Thymoma; **UCEC**: Uterine Corpus Endometrial Carcinoma.

Table S2: Clinicopathologic Features of APLN Expression in HCC patients from our Guangzhou cohort.

Variable (Guangzhou)	Low APLN expression (n = 46)	High APLN expression (n = 34)	*P* value
**Age**, mean ± SD	52.1 ± 13.0	56.0 ± 9.8	0.148
**Sex**			
Male	42 (91.3%)	31 (91.2%)	1.000
Female	4 (8.7%)	3 (8.8%)	
**TNM stage**			0.151
I	24 (52.2%)	8 (23.5%)	
II, III, IV	22 (47.8%)	26 (76.5%)	

NOTE. Values are n (%) unless otherwise indicated.

Table S3: Clinicopathologic Features of APLN Expression in HCC patients from TCGA.

Variable (TCGA)	Low APLN expression (n = 192)	High APLN expression (n = 138)	*P* value
**Age**, mean ± SD	60.0 ± 13.3	60.9 ± 12.1	0.542
**Sex**			
Male	125 (65.1%)	95 (68.8%)	0.554
Female	67 (34.9%)	43 (31.2%)	
**TNM stage**			0.098
I	103 (57.2%)	58 (45.3%)	
II	39 (21.7%)	39 (30.5%)	
III, IV	38 (21.1%)	31 (24.2%)	

NOTE. Values are n (%) unless otherwise indicated.

Table S4: Clinicopathologic Features of APLN Expression in HCC patients from GSE76427.

Variable (GSE76427)	Low APLN expression (n = 48)	High APLN expression (n = 67)	*P* value
**Age**, mean ± SD	64.2 ± 13.4	62.9 ± 12.2	0.611
**Sex**			
Male	41 (85.4%)	52 (77.6%)	0.343
Female	7 (14.6%)	15 (22.4%)	
**TNM stage**			0.850
I	22 (46.8%)	33 (49.3%)	
II, III, IV	25 (53.2%)	34 (50.7%)	

NOTE. Values are n (%) unless otherwise indicated.

Table S5. Primers used for quantitative PCR

ACTB-F	AGAGCTACGAGCTGCCTGAC
ACTB-R	AGCACTGTGTTGGCGTACAG
APLN-F	TGCTCTGGCTCTCCTTGAC
APLN-R	GCCCATTCCTTGACCCTCT
CCND1-F	CTGGAGGTCTGCGAGGAACA
CCND1-R	AGCTGCAGGCGGCTCTTT
CTNNB1-F	CATCTACACAGTTTGATGCTGCT
CTNNB1-R	GCAGTTTTGTCAGTTCAGGGA
**For CHIP-PCR**	
APLN-F	AGACCATAGGAGCTGTCCCA
APLN-R	ACCCTTCCGTGAGCAATTCA

Table S6. Relative cell viability of different cell lines treated with ML221.

ML221 (100 µM)	LO2				ML221 (100 µM)	HKCI-2			
day0	1.055	1.001	1.007	0.936	day0	1.001	1.039	1.028	0.932
day1	0.865	0.990	0.942	0.984	day1	0.535	0.527	0.539	0.500
day2	0.704	0.697	0.701	0.718	day2	0.276	0.270	0.256	0.236
day3	0.639	0.588	0.590	0.559	day3	0.137	0.124	0.130	0.147
day4	0.488	0.522	0.483	0.490	day4	0.125	0.138	0.132	0.125
ML221 (100 µM)	HepG2				ML221 (100 µM)	HKCI-10			
day0	1.040	1.026	0.977	0.957	day0	1.047	1.002	0.976	0.976
day1	0.660	0.709	0.709	0.729	day1	0.611	0.615	0.581	0.577
day2	0.485	0.487	0.473	0.489	day2	0.544	0.533	0.519	0.527
day3	0.438	0.410	0.402	0.412	day3	0.391	0.325	0.311	0.343
day4	0.335	0.383	0.381	0.382	day4	0.284	0.227	0.246	0.234
ML221 (100 µM)	PLC5				Cells were treated with 100 µM ML221 for different durations.				
day0	1.073	0.981	0.951	0.994					
day1	0.612	0.616	0.652	0.543					
day2	0.426	0.448	0.502	0.412					
day3	0.360	0.419	0.371	0.374					
day4	0.223	0.242	0.270	0.246					

ML-221 (µM)	LO2				ML-221 (µM)	HKCI-2			
0	0.964	1.085	0.920	1.031	0	1.115	1.076	0.972	0.837
1	1.064	1.173	0.995	1.046	1	0.853	0.727	0.806	0.932
10	1.018	0.959	0.987	1.054	10	0.566	0.716	0.566	0.597
100	0.639	0.588	0.590	0.559	100	0.137	0.124	0.130	0.147
ML-221 (µM)	HepG2				ML-221 (µM))	HKCI-10			
0	1.062	0.967	0.961	1.010	0	0.924	0.986	1.036	1.054
1	0.912	1.100	0.923	1.008	1	0.942	0.782	0.892	0.970
10	0.821	0.803	0.956	0.847	10	0.688	0.757	0.721	0.876
100	0.438	0.410	0.402	0.412	100	0.391	0.325	0.311	0.343
ML-221 (µM)	PLC5				Cells were treated with different concentrations of ML221 for three days.				
0	1.050	0.981	1.009	0.960					
1	0.934	0.933	0.922	0.918					
10	0.824	0.897	0.855	0.842					
100	0.360	0.419	0.371	0.374					
